# Inhibitory effects of *Lactobacillus casei* Shirota against both *Candida auris* and *Candida* spp. isolates that cause vulvovaginal candidiasis and are resistant to antifungals

**DOI:** 10.1186/s12906-021-03405-z

**Published:** 2021-09-23

**Authors:** Aline Lorenzoni Paniágua, Amabel Fernandes Correia, Lívia Custódio Pereira, Bruna Maciel de Alencar, Fabiana Brandão Alves Silva, Rosane Mansan Almeida, Yanna Karla de Medeiros Nóbrega

**Affiliations:** 1 Clinical Microbiology and Immunology Laboratory, Department of Pharmacy, University of Brasilia, Campus Darcy Ribeiro, Brasilia, DF 70910-900 Brazil; 2 Central Public Health Laboratory of the District Federal (LACEN-DF), Medical Biology Management, Center of Parasitology and Mycology, Brasília, DF Brazil; 3 Vulvar Pathology Clinic, Department of Gynecology, Brasilia University Hospital, University of Brasilia, Brasilia, DF Brazil

**Keywords:** Candidiasis, *Candida* spp., *Lactobacillus casei* Shiota, Microbicidal effect, Biofilm, *C. auris*

## Abstract

**Background:**

Vulvovaginal candidiasis (VVC), the second leading cause of genital infection in women of reproductive age, is caused by yeasts of the genus *Candida.* Treatment is usually empirical and performed with azoles, which have shown increasing ineffectiveness due to resistance from these species. This therapeutic challenge has led to the search for new treatment strategies. *Lactobacillus* spp. produce several components with microbicidal effects, such as lactic acid. These species are the main components of a healthy vaginal microbiota and have been used as probiotics. The aim of this work was to investigate the in vitro inhibitory effects of *Lactobacillus casei* Shirota on both the *Candida* spp. that cause VVC and on *C. auris*.

**Methods:**

The microbicidal effects of *L. casei* Shirota on the main VVC-causing species, *C. albicans*, *C. tropicalis*, *C. norvegensis* and *C. parapsilosis,* in addition to *C. auris* were investigated by counting the Colony-forming Units (CFUs) after cocultivation. The antifungal activity of lactic acid against these *Candida* strains was assessed using the microtiter broth dilution method to determine the minimum inhibitory concentrations (MICs). The effects of *L. casei* Shirota on hyphal and early biofilm formation was measured by optical microscopy.

**Results:**

*L. casei* Shirota showed inhibitory action against all tested *Candida* spp., ranging from 66.9 to 95.6% inhibition depending on the species. This inhibition is possibly related to the production of lactic acid, since lactic acid has shown microbicidal action against these same *Candida* spp. at a concentration of 5 mg/mL, which corresponds to half of the normal physiological concentration. In addition, *L. casei* Shirota was able to reduce the formation of *C. albicans* hyphae and early biofilms, showing strong anti-*Candida* effects.

**Conclusions:**

These results suggest that *L. casei* Shirota has antifungal activity against the *Candida* species that cause VVC. *L. casei* also has microbicidal action against *C. auris*.

## Background

Vulvovaginal candidiasis (VVC) is the second leading cause of genital infection in women after only bacterial vaginosis [1]. VVC affects up to 78% of women of reproductive age [2], impairing their quality of life [3]. The main causative agent is *Candida albicans*, although there has been a recent increase in the incidence of VVC cases caused by other species, such as *Candida glabrata, Candida tropicalis, Candida krusei, Candida parapsilosis* and *Candida guilliermondii* [4, 5].

In clinical practice, treatment is usually empirical, and fluconazole is the drug of choice [6]. However, an increasing proportion of *Candida* strains are resistant to this treatment [7]. This resistance, in addition to the toxicity of some azoles [8, 9], has led to the search for alternative therapies to treat VVC.

Anti-*Candida* properties, such as the suppression of inflammatory genes [10] and the reduction in adherence and biofilm formation [11, 12], have been attributed to *Lactobacillus* strains. The production of several substances with microbicidal action – such as acetic acid, bacteriocins, biosurfactants, hydrocarbons, hydrogen peroxide, coaggregation molecules [13] and lactic acid – have also been reported. Among them, lactic acid is considered the most important postbiotic substance [14].

The vaginal microbiota plays an important role in preventing infections. *Lactobacillus* spp. are predominant in approximately 70% of healthy women [15], but some species may not play a protective role [16]. The intestine is a reservoir for microorganisms that access the lower genital tract through the perianal region [17]. Thus, oral administration of *Lactobacillus* probiotics alters the intestinal microbiota of the rectum and consequently may alter the vaginal microbiota. This strategy can be a useful measure to treat VVC. Although *L. casei* Shirota is one of the most commonly consumed probiotic strains worldwide, to the best of our knowledge, there have been no studies regarding its action against *Candida* strains involved in VVC. The aim of this work was to investigate the inhibitory effects of *L. casei* Shirota against strains of *Candida* spp. isolated from VVC cases and also the effects of *L. casei* Shirota against *C. auris*.

*C. auris* is an emerging multiresistant pathogen that causes severe infections for which treatment resources are scarce [18]. An *L. paracasei* strain was recently shown to have both probiotic and postbiotic action against *C. auris* in an in vivo model [19]. Moreover, depending on the method employed, *C. auris* can be misidentified as *C. parapsilosis* or *C. guilliermondii*, two VVC-causing species [18].

## Methods

### Origin, isolation and maintenance of the strains

*Candida* spp. isolates were obtained from patients examined at the Gynecology Outpatient Service of the University of Brasilia Hospital (HUB) and identified at the Central Public Health Laboratory of the Federal District (LACEN-DF). We sought and obtained individual written informed consent from all subjects. For this study, clinical isolates of *C. albicans, C. glabrata, C. tropicalis, C. krusei, C. parapsilosis* and *C. norvegensis* that showed resistance to one or more antifungals were selected. The multiresistant strain of *C. auris* used in this study was kindly provided by LACEN-DF. *Candida* cells were routinely maintained on Sabouraud dextrose agar (1% peptone, 4% dextrose and 1.5% agar) or YM broth (1% glucose, 0.3% malt extract, 0.5% peptone and 0.3% yeast extract) [20]. The *Lactobacillus casei* Shirota strain was isolated from fermented milk (*L. casei* Shirota, Yakult®, lot number H1336) and maintained in MRS medium (1% peptone, 1% meat extract, 0.5% yeast extract, 2% dextrose, 0.5% sodium acetate, 0.1% polysorbate 80, 0.2% potassium phosphate, 0.2% ammonium citrate, 0.01% magnesium sulfate and 0.005% manganese sulfate) [21].

The isolates were identified by matrix-assisted laser desorption ionization time-of-flight (MALDI-TOF) mass spectrometry. Briefly, a few colonies were applied to the target slide (Biomérieux, Marcy l’Etoile, France) with 0.5 μL of 25% formic acid and 1 μL of 3.1% matrix solution (alpha-cyano-4-hydroxycinnamic acid) (both from Biomérieux, MarcyI’Etoile, France). After drying, the slides were transferred to a Vitek MS® System reading station (Biomérieux, Marcy l’Etoile, France), and the obtained spectra were analyzed using the Vitek MS Version 3.0 database. The results were considered valid when the percent probability of identification values were greater than or equal to 99.9% [22].

### Fungicidal activity in coculture

*L. casei* Shirota/*Candida* spp. cocultures were generated according to Kang et al. [21] with modifications. First, *L. casei Shirota* and *Candida* spp. strains were grown separately in MRS broth at 37 °C for 24 h and in YM broth at 37 °C for 18–24 h, respectively. For cocultures, both *L. casei* Shirota and *Candida* strains were equally (1:1) inoculated in tubes containing 5 mL of mixed YM/MRS broth (v/v) and incubated at 37 °C for 24 h. The starting inocula - 1 × 10^8^ cells each - were adjusted to an OD600 (optical density at 600 nm) in a volume of 5 to 20 μL. The pH was measured prior to and after incubation of the cocultures. *Candida* spp. strains (1 × 10^8^ cells) were incubated by themselves (monocultures) for use as positive controls. Non-inoculated medium was used as a negative control. After growth, 10 μL of the cocultures were diluted 1:20, seeded in Petri plates containing YM and MRS media and incubated at 37 °C for 24 h. Determination of the microbicidal activity of *L. casei* against *Candida* species was assessed by counting the number of colony-forming units per milliliter (CFUs/mL). All samples were tested in technical and biological triplicates (independent experiments).

To evaluate the fungicidal activity of *L. casei* Shirota, the number of CFUs observed in the *Candida* spp. monocultures (control) were compared to those in the cocultures. Statistical significance of the difference in CFUs between the cocultures and controls was analyzed using Student’s t-test. The percentage of microbicidal activity (%) was calculated employing the following formula:

$$ \%\mathrm{microbicidal}\ \mathrm{activity}=\left(\mathrm{CFU}\ \mathrm{cocultivation}\times \kern0.37em 100/\mathrm{CFU}\ \mathrm{positive}\ \mathrm{control}\right)-100 $$

### Dosage of lactic acid

The lactic acid measurement was performed in the cocultivation broths prior to and after 24 h of incubation. A filtration step was performed using a 0.2 μm filter before the measurements, and the dosage was determined by colorimetry with Cobas 6000 equipment (Roche Diagnostics).

### Minimal inhibitory concentration (MIC) of lactic acid by microdilution

A microdilution assay was performed in 96-well plates with a volume of 200 μL/well and 1 × 10^6^ fungal cells [20]. Serial dilutions of a 2% DL-lactic acid (Sigma-Aldrich, 69,785) solution were performed to obtain working solutions of 10, 5 and 2.5 mg/mL. The microplate was incubated at 35 °C for 24 h, and 10 μL of the content of each well was seeded in YM plates and incubated at 37 °C for 24 h. All samples and controls were tested in technical triplicates and biological duplicates (independent experiments).

### Effects of *L. casei* Shirota on the yeast-to-hyphae transition and early biofilm formation

To evaluate the effects of *L. casei* Shirota on the yeast-to-hyphae transition and initial biofilm formation of *Candida* spp., cocultures of *L. casei/Candida* were grown in 96-well flat-bottom polystyrene plates. The experiment was carried out using clinical isolates and the reference *C. albicans* strains ATCC 90028 and SC5314. In brief, 100 μL/well RPMI 1640 medium was supplied with serum 10% (v/v) and inoculated with a cell suspension of 1 × 10^6^ yeast and 1 × 10^8^ bacteria per milliliter. The plates were incubated at 37 °C for 24 h to obtain early biofilms. Images were captured with either a Nikon Eclipse E200 or an Mrm CCD camera (Carl Zeiss GmbH), and the microscope and camera were jointly operated for image capture by Zen 2012 software with 10× and 40× objectives.

### Statistical analysis

Statistical analysis was performed by Student’s t-test, considering values of ≤0.05 as statistically significant after comparing the tested samples and controls.

## Results

### Anti-*Candida* activity of *L. casei* Shirota

**Fig. 1:**
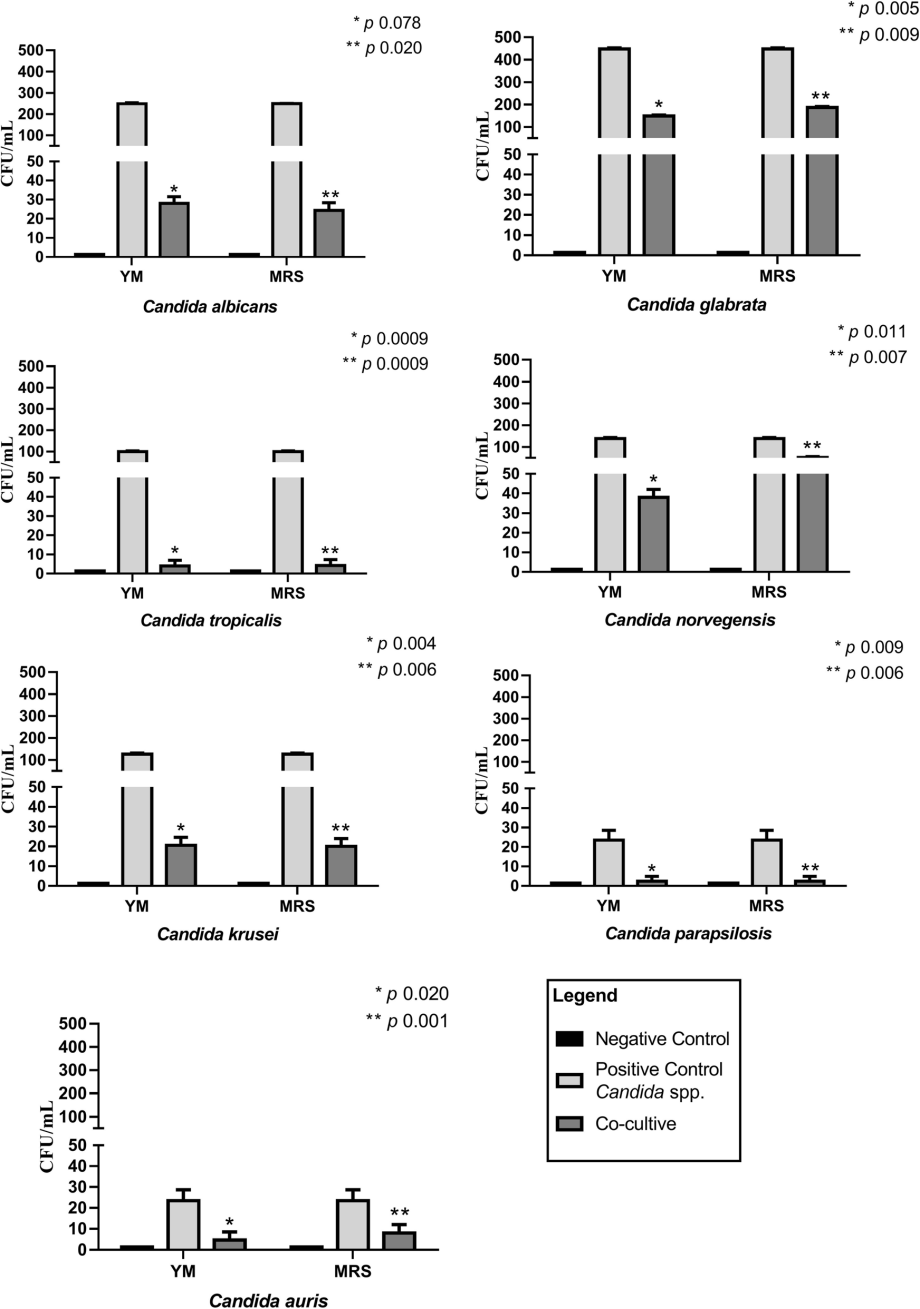
Microbicidal activity of *L. casei* Shirota against *Candida* species. Evaluation of the microbicidal action after cocultivation in YM and MRS agar medium (CFUs/mL)

The *L. casei* Shirota strain showed high microbicidal activity against all tested fungal species, inhibiting their growth after 24 h of coculture. Figure 1 shows the CFU/mL counts in YM medium before (monoculture) and after coculture. We obtained the following reduced CFU counts: *C. albicans*, from 249.6 to 27.6 CFU/mL (9×); *C. glabrata*, from 447.3 to 149.3 CFU/mL (3×), *C. tropicalis*, from 99.3 to 4.0 CFU/mL (24×), *C. norvegensis*, from 138.6 to 38, 6 CFU/mL (3.5×), *C. krusei*, from 126.6 to 20.6 CFU/mL (6×), *C. parapsilosis*, from 23.6 to 1.3 CFU/mL (18×) and *C. auris*, from 23.6 to 4.6 CFU/mL (5×). Thus, inhibition by *L. casei* Shirota was most effective against *C. tropicalis* and least effective against *C. auris* after analysis in YM medium.

To verify the influence of the medium on the growth inhibition of *Candida* spp., CFU counts were also performed using MRS medium, which favors the exuberant growth of *Lactobacillus* spp. while also allowing the growth of yeasts. We obtained the following reduced CFU counts: *C. albicans* was reduced from 249.6 to 24.3 CFU/mL (10×); *C. glabrata*, from 447.3 to 187.3 CFU/mL (2.4×); *C. tropicalis*, from 99.3 to 4.3 CFU/mL (23×); *C. norvegensis*, from 138.6 to 52.6 CFU/mL (2.6×); *C. krusei*, from 126.6 to 20 CFU/mL (6.3×); *C. parapsilosis*, from 23.6 to 1.3 CFU/mL (18×); and *C. auris*, from 23.6 to 8.0 CFU/mL (3×). Once again, growth inhibition was most effective against *C. tropicalis* and least effective against *C. auris*. There was no significant difference in the antifungal activity of *L. casei* in the two types of culture media used (Fig. 1). These data confirm the reduction in CFUs/mL of all tested *Candida* species after coculture with *L. casei* Shirota (Fig. 1).

The percent microbicidal activity of *L. casei* Shirota against *Candida* spp. strains (%) was calculated by setting the positive control as 100% growth. The microbicidal activities verified in YM medium as percentages were *C. albicans* 89.2%, *C. glabrata* 66.9%, *C. tropicalis* 95.6%, *C. norvegensis* 62.7%, *C. krusei* 87.0%, *C. parapsilosis* 90.1% and *C. auris* 86.6% (Table 1). The results obtained in MRS medium were as follows*: C. albicans* 93.8%, *C. glabrata* 69.1%, *C. tropicalis* 95.2%, *C. norvegensis* 67.3%, *C. krusei* 85.0%, *C. parapsilosis* 94.8% *and C. auris* 93.9% (Table 1).

**Table 1:** Percent inhibition of *Candida* growth after cocultivation with *L. casei* Shirota

	PC ***Candida*** spp. (N)	YM		MRS	
		Cocultivation ***Candida*** spp***. + L. casei*** (N)	Microbicidal activity (%)	Cocultivation ***Candida*** spp***. + L. casei*** (N)	Microbicidal activity (%)
***Candida albicans***	249.6	27.6	89.2	24.3	90.8
***Candida glabrata***	447.3	149.3	66.9	187.3	69.1
***Candida tropicalis***	99.3	4.0	95.6	4.3	95.2
***Candida norvegensis***	138.6	38.0	62.7	52.6	67.3
***Candida krusei***	126.6	20.6	87.0	20.0	85.0
***Candida parapsilosis***	23.6	1.3	90.1	1.3	94.8
***Candida auris***	23.6	4.6	86.6	8.0	93.9

*PC* (Positive control) : *Candida* spp. monocultures corresponding to 100% growth

Although the inhibition spectra varied depending on the culture medium and the species involved, in all cocultures, the activity was greater than 60% and reached as high as 95.6%. For *C. auris*, the microbicidal activity reached 93.9%.

### Production of lactic acid in the cocultures

The pH values in the culture medium were evaluated prior to and after the 24 h incubation period. In all cocultures, the pH measured ranged from 6.0 to 5.0 after 24 h, while no change in pH was observed for cultures of *Candida* isolated in YM medium.

The lactic acid production of the cocultures was then measured. The values obtained for cocultivation were subtracted from the baseline values obtained for *Candida* strains. Noninoculated YM and MRS media were used as negative controls. Lactic acid production was observed in all evaluated cocultures. The production ranged from 6.51 to 8.24 mg/mL, which is slightly lower than the 10.34 mg/mL obtained for *L. casei* grown by itself (Table 2).

**Table 2:** Lactic acid production by *L. casei* Shirota and minimum inhibitory concentrations of lactic acid

	Lactic acid production (mg/mL)		MIC
	Monocultures (***Candida*** spp.)	Cocultures (+ ***L. casei)***	Lactic acid (mg/mL)
**YM (NC)**	0	0	NA
**MRS (NC)**	0	0	NA
**YM + MRS (NC)**	0	0	NA
***Lactobacillus casei*(PC)**	10.34	NA	NA
***Candida albicans***	0.020	7.07	2.5
***Candida glabrata***	0.019	6.51	2.5
***Candida tropicalis***	0.021	7.58	2.5
***Candida norvegensis***	0.025	8.22	2.5
***Candida krusei***	0.019	7.80	2.5
***Candida parapsilosis***	0.023	8.14	5.0
***Candida auris***	0.021	8.24	2.5

*YM (NC)*: YM medium uncultivated/negative control, *MRS (NC)*: MRS medium uncultivated/negative control, *YM + MRS (NC)*: Mixed YM and MRS media uncultivated/negative control, *PC*: Positive control, *NA*: Not applicable

### Minimal inhibitory concentration (MIC) of lactic acid

To confirm the action of lactic acid on the inhibition of *Candida* growth, three different concentrations (10, 5 and 2.5 mg/mL) of lactic acid were evaluated. These concentrations were chosen based on the biological production of *Lactobacillus* present in the normal healthy microbiota, where the concentration is approximately 10 mg/mL (1%) [23].

Table 2 shows the minimal inhibitory concentrations (MICs) of lactic acid against the *Candida* spp. The lowest tested concentration, 2.5 mg/mL, had an antimicrobial effect against most *Candida* species, except for *C. parapsilosis*, whose inhibitory concentration was found to be 5 mg/mL. Considering an MIC of 5 mg/mL (50% of the physiological concentration), lactic acid showed antimicrobial activity against all tested *Candida* strains. Note that all of the *Candida* strains in this experiment had previously shown fluconazole resistance.

### *L. casei* Shirota impairs the yeast-to-hyphae transition in *C. albicans*

**Fig. 2:**
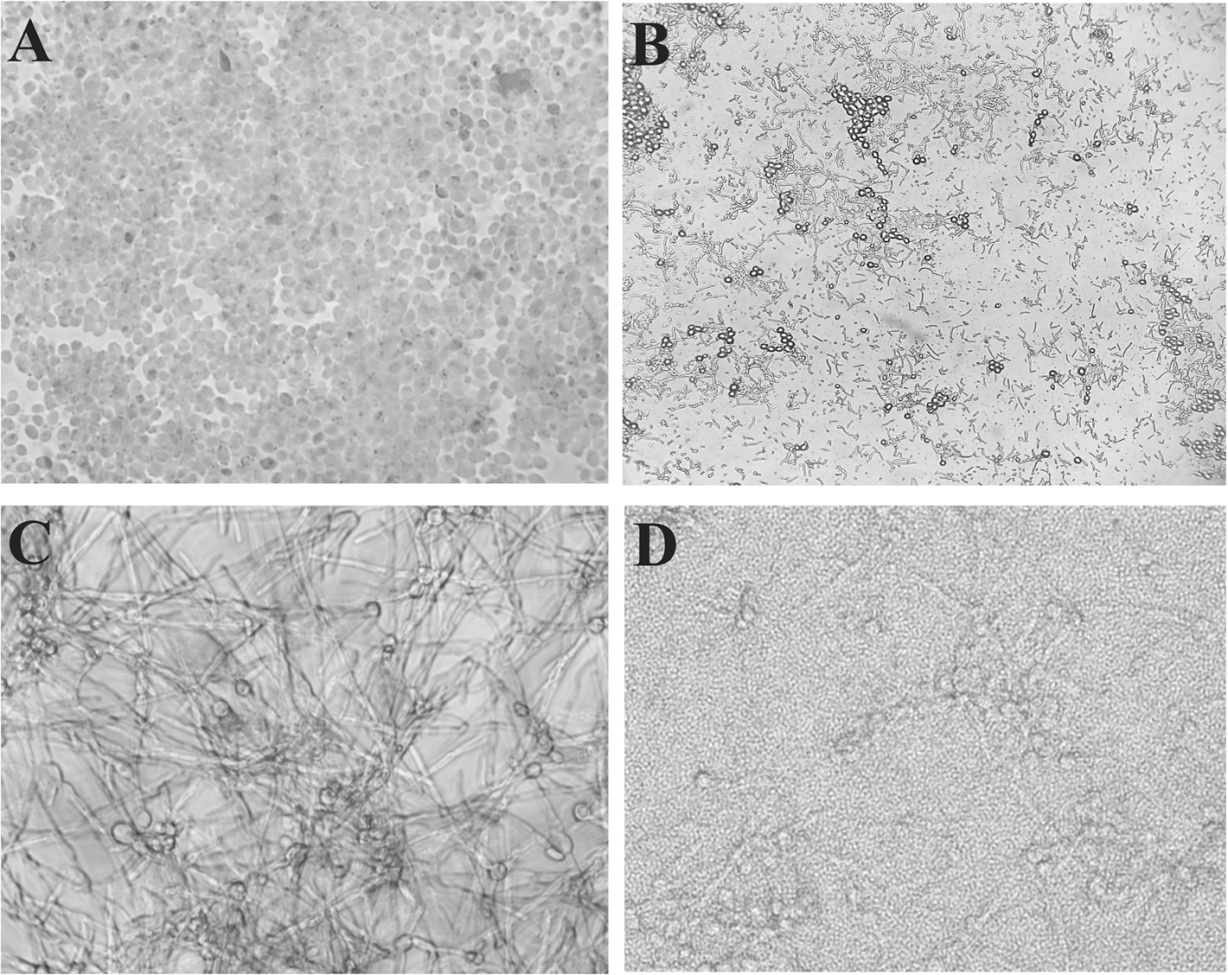
Effects of *L. casei* Shirota on the early biofilm formation and hyphal growth of *Candida* spp. *C. albicans* 90028 (**A**) and SC5314 (**C**) incubated in the presence of serum (10%) at 37 °C for 24 h. Few yeast cells were seen after cocultivation with *L. casei* Shirota (**B**). Hyphae formation was strongly impaired when *Candida* strains were cocultured with *L. casei* Shirota (**D**). Magnification 1000× (**A**, **C** and **D**); **B** 400 ×

There has been a consensus that the yeast-to-hyphae transition is a crucial factor in *C. albicans* biofilm formation and virulence [24]. Consequently, we decided to investigate the effects of *L. casei* cocultivation on *C. albicans* filamentous growth. Therefore, yeasts were cultivated in the presence of fetal bovine serum (FBS) to allow the formation of hyphae. The SC5314 strain was used due to its recognized ability to quickly form filaments [25]. By microscopic analysis, both *C. albicans* ATCC90028 and *C. albicans* SC5314 properly produced germ tubes after 3 h of induction (data not shown). After 24 h, long hyphae were observed in only the controls (Fig. 2C). On the other hand, when in coculture with *L. casei*, the yeast-to-hyphae transition was strongly impaired compared to the controls, and only a few pseudohyphae and blastoconidia were observed (Fig. 2D).

### *L. casei* Shirota reduces the initial adhesion of *Candida* biofilms

Initial adhesion to a surface is the first step in biofilm formation. Since *L. casei* Shirota impaired the yeast-to-hyphae transition, we decided to evaluate the effects of this strain on the initial biofilm formation of *Candida* strains. After 24 h, a strong and consistent cell layer covering the whole surface was observed in the control sample (Fig. 2A). On the other hand, in the cocultures, the presence of *L. casei* caused a clear reduction in fungal growth, and empty spaces among the few yeast cells remaining as well as many *L. casei* Shirota cells were observed (Fig. 2B).

Due to the three-dimensional nature of biofilms and the use of optical microscopy, we were not able to visualize fully formed biofilms. However, we demonstrated that *L. casei* Shirota is capable of interfering with the initial adhesion of *Candida* cells.

## Discussion

VVC is one of the most frequent diagnoses in gynecology and the second most common genital infection globally [1]. One of the main problems associated with these infections is the increasing rate of antifungal therapy failure [7], which leads to episodes of recurrent VVC [17]. This increasing resistance has motivated the search for alternative therapies to control VVC, and the use of *Lactobacillus* spp. as probiotics is one of the most promising strategies [26].

In this study, *L. casei* Shirota showed inhibitory activity against all tested *Candida* strains, which represent the main etiological agents of VVC [5], in addition to the emerging pathogen *C. auris*. Inhibition by *L. casei* was very effective (89.2%) on the most prevalent species, *C. albicans* [17], and 95.6% inhibition was reached against *C. tropicalis*.

Growth inhibition is probably related to the production of lactic acid since the microdilution tests showed that *Candida* spp. do not grow at a lactic acid concentration of 5 mg/mL. This result corroborates recent studies that indicate that the production of lactic acid is the main microbicidal factor associated with strains of *Lactobacillus* [14, 27].

*L. casei* Shirota was also able to reduce the formation of *C. albicans* hyphae. Many studies support a strong link between hyphal morphogenesis and *C. albicans* pathogenicity [24]. In a mouse model of systemic infection, mutant *C. albicans* was unable to switch its form, resulting in significantly reduced virulence [28]. Moreover, the yeast-to-hyphal transition plays a pivotal role in the transition from commensalism to pathogenicity [29]. In patient tissue samples, *C. albicans* was found predominantly in the hyphal form [24]. Furthermore, hyphal formation was shown to contribute to the ability of *C. albicans* cells to kill macrophages [30].

Based on our initial data, we hypothesized that *L. casei* Shirota could also have some effect on the formation of early biofilm structures, probably because hyphae were not properly produced. Biofilm structures enable microorganisms to colonize and exacerbate clinical infections through dissemination into the bloodstream [31], leading to invasive systemic infections, which are extremely difficult to eradicate because of their high resistance to antifungal drugs [32].

The primary source of vaginal microbiota is the intestine [33]. After oral administration, probiotic strains of *Lactobacillus* have already been demonstrated to survive and disperse in the vagina in some studies. Joo [34] demonstrated that *L. helveticus* administered orally or vaginally reduced the growth of *Candida* in immunosuppressed mice. Falagas [35] reviewed this subject and found evidence in favor of oral supplementation with lactobacilli to prevent VVC. Due to its inhibitory actions against growth and hyphae and early biofilm formation in *Candida*, *L. casei* Shirota could be an interesting alternative for the management of VVC. A next step would be to perform in vivo trials to confirm this hypothesis.

## Conclusion

This work presents evidence that *L. casei* Shirota derived from fermented milk has microbicidal action against *Candida albicans* as well as on non-*C. albicans* strains, which have been found with increasing frequency as etiologic agents of VVC. *L. casei* Shirota was also shown to have a microbicidal effect on *C. auris*, a multidrug-resistant species whose recent emergence is of worldwide concern.

## Data Availability

The datasets used and/or analyzed during the current study are available from the corresponding author on reasonable request.

## Abbreviations

VVC: Vulvovaginal candidiasis
MIC: Minimum inhibitory concentration
MALDI-TOF: Matrix-Assisted Laser Desorption/Ionization Time of Flight
MRS: Man, Rogosa and Sharpe
YM: Yeast and Mold
CFU: Colony-forming units
ATCC: American Type Culture Collection
